# Indonesian state educational universities’ bibliometric dataset

**DOI:** 10.1016/j.dib.2018.11.128

**Published:** 2018-11-30

**Authors:** Lantip Diat Prasojo, Reni Fatmasari, Eti Nurhayati, Ahmad Darmadji, Fitri Ayu Kusumaningrum, Yuli Andriansyah

**Affiliations:** a Department of Educational Management, Postgraduate Program, Universitas Negeri Yogyakarta, Indonesia; b Faculty of Education, Monash University, Australia; c Department of Islamic Early Childhood Education, Faculty of Islamic Education, Institut Agama Islam Negeri Syekh Nurjati, Indonesia; d Department of Islamic Education & Master of Islamic Studies, Faculty of Islamic Studies, Universitas Islam Indonesia, Indonesia; e Department of Psychology, Faculty of Psychology and Socio-Cultural Studies, Universitas Islam Indonesia, Indonesia; f Department of Islamic Economics, Faculty of Islamic Studies, Universitas Islam Indonesia, Indonesia

## Abstract

This data article presents an important bibliometric dataset of ten Indonesian leading educational universities. These ten Indonesian leading educational universities are Universitas Pendidikan Indonesia (UPI), Universitas Negeri Malang (UM), Universitas Negeri Semarang (UNNES), Universitas Negeri Surabaya (Unesa), Universitas Negeri Yogyakarta (UNY), Universitas Negeri Makassar (UNM), Universitas Negeri Jakarta (UNJ), Universitas Negeri Padang (UNP), Universitas Negeri Medan (Unimed), and Universitas Negeri Gorontalo (UNG). Using Scopus database search analysis, this data article collects data on documents per year, subject area of documents, source title of documents, documents type, country/territory, author name and number of documents, affiliation and most cited documents for each universities.

**Specifications table**

**Table:** 

Subject area	Education
More specific subject area	Higher education
Type of data	Table and figure
How data was acquired	Scopus search analysis
Data format	Raw and filtered
Experimental factors	Institutional search in Scopus is conducted to obtain ten Indonesian leading educational universities
Experimental features	Search analysis in Scopus is conducted for each ten Indonesian leading educational universities to obtain bibliometric data
Data source location	Indonesia
Data accessibility	The dataset is accessible within this article data
Related research article	1. A. Darmadji, L. D. Prasojo, Y. Riyanto, F. A. Kusumaningrum, and Y. Andriansyah, “Publications of Islamic University of Indonesia in Scopus Database: a bibliometric assessment,” *COLLNET J. Sci. Inform. Manag.*, **12** (1), 2018, 109–131. 2. A. Darmadji, L. D. Prasojo, F. A. Kusumaningrum, and Y. Andriansyah, “Research productivity and international collaboration of top Indonesian universities,” *Curr. Sci.*, **115** (4), 2018, 653–658.

**Value of the data**

• The dataset in this article will help further research on research quality of Indonesian educational universities. Policy makers, researchers, and other stakeholders can use the dataset to compare research quality of each university.
• The dataset will also provide useful tool for analysis in university level. Policy makers, researchers, and other stakeholders can use the dataset to analyze research quality of each university in terms of productivity, collaboration, citation and others.
• The dataset will also be useful in conducting citation analysis of highly-cited documents. This particular data can provide insight on how to publish in high impact academic journals as well as conferences.

## 1 Data

This article data presents bibliometric dataset of ten Indonesian leading educational universities. Educational universities in this context are those that previously known as IKIP or Institut Keguruan dan Ilmu Pendidikan (Institute of Teaching and Educational Sciences). These universities previously provide higher education services to prepare teachers all over Indonesia after Independence Day. In the late 1990s and early 2000s, Government of Indonesia transform these universities into more open in educational services by providing courses in education, law, humanities, business, engineering, technology and others. This transformation changes also these universities name from previously institute to university.

After the transformation, some of educational universities show its capacity to compete to improve research quality. This dataset focuses on leading educational universities with largest publication in Scopus. Scopus is chosen as database because of its usage by Government of Indonesia to rank universities. Ranking these Indonesian leading educational universities based on Scopus documents results in names as follow: Universitas Pendidikan Indonesia (UPI), Universitas Negeri Malang (UM), Universitas Negeri Semarang (UNNES), Universitas Negeri Surabaya (Unesa), Universitas Negeri Yogyakarta (UNY), Universitas Negeri Makassar (UNM), Universitas Negeri Jakarta (UNJ), Universitas Negeri Padang (UNP), Universitas Negeri Medan (Unimed), and Universitas Negeri Gorontalo (UNG).

Table 1 below summarizes ten Indonesian leading educational universities name and its abbreviation, documents in Scopus, first year in Scopus, average documents until 2010, and average documents from 2011 to 2018. This data is useful to understand the pattern of these universities’ documents in Scopus database. Average documents before and after 2010 are presented to show the increasing trend in documents in Scopus as implication of policies by Government of Indonesia regarding Scopus. In 2010s, Government of Indonesia uses Scopus for some important policies for higher education: 1) document in Scopus is required for professor tenure[1]; 2) university ranking based on document in Scopus [2], [3]; and 3) researcher ranking based on document and citation in Scopus [2]. These policies imply competition among universities, including educational ones, to publish in Scopus-indexed journals and conference that make improvement in data in Table 1 reasonable.

**Table 1:** General information of ten Indonesian educational universities in dataset.

**University Name and Abbreviation**	**Documents in Scopus**	**First Year in Scopus**	**Average Documents Until 2010**^a^	**Average Documents 2011-2018**^a^
Universitas Pendidikan Indonesia (UPI)	1382	2002	4.22	168.00
Universitas Negeri Malang (UM)	611	2000	6.55	67.38
Universitas Negeri Semarang (UNNES)	559	2001	2.00	67.38
Universitas Negeri Surabaya (Unesa)	547	2000	1.45	66.38
Universitas Negeri Yogyakarta (UNY)	340	2002	1.78	40.50
Universitas Negeri Makassar (UNM)	338	2000	0.91	41.00
Universitas Negeri Jakarta (UNJ)	304	2001	2.20	35.25
Universitas Negeri Padang (UNP)	261	2003	1.25	31.38
Universitas Negeri Medan (Unimed)	141	2000	1.55	15.50
Universitas Negeri Gorontalo (UNG)	104	2008	0.67	12.75

a Authors estimation.

After using Scopus database to search for leading educational universities in Indonesia, further step is collecting data on documents per year, subject area of documents, source title of documents, documents type, country/territory, author name and number of documents, affiliation and most cited documents for each universities. This data were then saved in an .xlsx file for each university. Every .xlsx file consists of data for single university. To explain what each .xlsx file consists of, sample of Universitas Pendidikan Indonesia will be described in the following tables and figure.

Table 2 below describes number of documents per year in Scopus published by Universitas Pendidikan Indonesia (UPI). This table informs initial year the university published document in Scopus and its development until recently. Increasing trend in data can also simply be viewed from this table.

**Table 2:** Documents per Year of Universitas Pendidikan Indonesia (UPI).

**Year**	**Documents**
2002	1
2003	5
2004	0
2005	5
2006	5
2007	3
2008	5
2009	0
2010	14
2011	13
2012	28
2013	41
2014	50
2015	55
2016	270
2017	507
2018	380

**Fig. 1:**
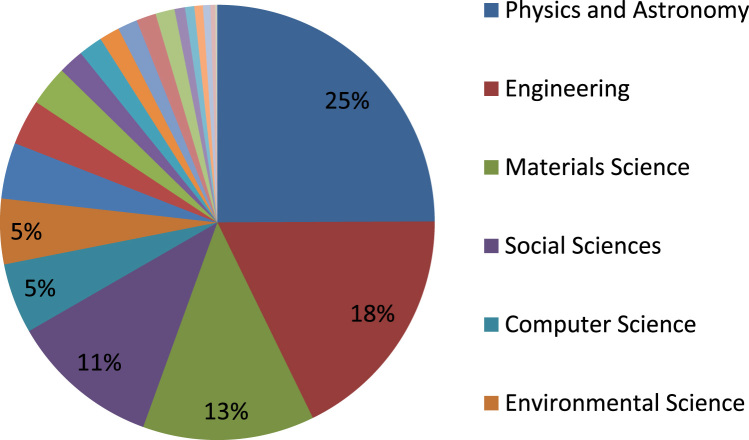
Subject Area of Documents of Universitas Pendidikan Indonesia (UPI).

Fig. 1 below describes subject area of documents by Universitas Pendidikan Indonesia (UPI) in Scopus. This subject area can inform the strength of the university in publication. It can also be a good source to evaluate transformation of the university from previously focused on education to a wider range of teaching as today. Policy makers from Government of Indonesia can use this data to analyze whether policy on educational university has a shift paradigm in publication and soon.

Table 3 below describes source titles of documents by Universitas Pendidikan Indonesia (UPI) in Scopus. The data on source title can inform the trend in the university’s publication. A researcher can use this data to analyze quality of source based on its impact factor, SCIMago journal ranking, CiteScore, and other measurements. The source can also be a good source to determine the university policy in publication: whether it focuses on quantity, by allowing researchers to publish at any kind of Scopus-indexed journals and conferences, or quality, by emphasizing on only high impact journals or conferences.

**Table 3:** Source Title of Documents of Universitas Pendidikan Indonesia (UPI).

**Source Title**	**Documents**
Journal of Physics Conference Series	392
IOP Conference Series Materials Science And Engineering	243
AIP Conference Proceedings	127
IOP Conference Series Earth And Environmental Science	62
Indonesian Journal of Applied Linguistics	44
Jurnal Pendidikan IPA Indonesia	31
Man In India	31
Pertanika Journal of Science And Technology	19
International Journal of Applied Business And Economic Research	16
Advanced Science Letters	11
International Journal Of Economic Research	11
Journal Of Engineering Science And Technology	11
ARPN Journal Of Engineering And Applied Sciences	6
New Educational Review	6
American Journal Of Applied Sciences	5
Indonesian Journal Of Chemistry	5
International Journal Of Instruction	5
Jurnal Teknologi Sciences And Engineering	5
Materials Science Forum	5
Social Sciences Pakistan	5

Table 4 below describes document types of publication in Scopus by Universitas Pendidikan Indonesia (UPI). The data can inform the university policy in publishing research. Government of Indonesia considers article has a greater value than conference paper. Thus, when the university emphasizes on conference paper, it can be an indication for its effort to improve number of documents. Document types can also inform the variety of academic communication by the university.

**Table 4:** Document Types of Universitas Pendidikan Indonesia (UPI).

**Document Type**	**Documents**
Conference Paper	949
Article	400
Book Chapter	11
Review	7
Editorial	6
Article in Press	4
Erratum	3
Note	2

Table 5 below describes country/territory of document origin by Universitas Pendidikan Indonesia (UPI) in Scopus. The data can inform collaboration pattern of the university in term of country. The data shows Indonesia in top list which can be interpreted as main source of document origin. Japan follows in second list as indication of 63 documents published in collaboration with researchers from this country. This data will also be useful to map collaboration based on regions. Researchers for example can analyze publications in collaboration with Asian, European, American countries, and soon.

**Table 5:** Country/Territory of Documents of Universitas Pendidikan Indonesia (UPI).

**Country/Territory**	**Documents**
Indonesia	1379
Japan	63
Malaysia	32
South Korea	19
Netherlands	16
Australia	15
United States	15
Turkey	8
China	7
Pakistan	7
Poland	7
Saudi Arabia	7
Canada	6
Italy	6
Mexico	6
Brazil	5
Bulgaria	5
Croatia	5
Estonia	5
Ghana	5
Hong Kong	5
Hungary	5
India	5
Iran	5
Kenya	5
Nigeria	5
Portugal	5
Romania	5
Russian Federation	5
Slovakia	5
Spain	5
Uganda	5
United Kingdom	5
Philippines	4
Switzerland	4
France	3
Germany	3
Singapore	3
Bosnia and Herzegovina	2
Colombia	2
Israel	2
Taiwan	2
Thailand	2
Argentina	1
Austria	1
Chile	1
Czech Republic	1
Finland	1
Norway	1
Peru	1
Serbia	1
Ukraine	1
Undefined	3

Table 6 below describes prominent authors in Universitas Pendidikan Indonesia (UPI). These authors are those whose largest documents in Scopus. The data on these authors can be useful to analyze disparities among staff member of university. Policy makers can also use the data as source of potential individuals tasked to improve university publication. For public, these authors can also be sources for commentary and analysis for occurring events in the society.

**Table 6:** Authors of Documents of Universitas Pendidikan Indonesia (UPI).

**Author Name**	**Documents**
Nandiyanto, A.B.D.	70
Abdullah, A.G.	51
Suhandi, A.	38
Rustaman, N.Y.	31
Samsudin, A.	28
Hasanah, L.	27
Setiawan, A.	27
Jupri, A.	26
Firman, H.	24
Kaniawati, I.	24
Suryadi, D.	22
Permanasari, A.	20
Riza, L.S.	20
Rusdiana, D.	20
Hidayat, T.	19
Khoerunnisa, F.	19
Liliasari	19
Redjeki, S.	19
Munir	18
Herman, T.	17

Table 7 below describes affiliations in documents by Universitas Pendidikan Indonesia (UPI) in Scopus. The data consists of universities, research institutions, libraries and other entities having collaboration with the university in publishing documents. This type of data can be very useful to analyze collaboration pattern in research in terms of internationalization. Government of Indonesia has encouraged researchers to collaborate with potential partners especially in developed countries by providing relevant grants. Such data can be resource to track the results of this policy. Furthermore, having collaboration with world leading universities, for example those that are listed in Top 100 QS World University Rankings, will also has significant value for accreditation. Thus, policy makers in university can use the data to plan further potential collaboration and choose potential university partners.

**Table 7:** Affiliation in documents by Universitas Pendidikan Indonesia (UPI).

**Affiliation**	**Documents**
Universitas Pendidikan Indonesia	1382
Institut Teknologi Bandung	123
Universitas Padjadjaran	23
Hiroshima University	21
UIN Sunan Gunung Djati	14
Universitas Sultan Ageng Tirtayasa	14
Universitas Riau	11
State University of Malang	11
Shinshu University	10
Universitas Sriwijaya	10
Universitas Muhammadiyah Prof. Dr. HAMKA	10
Universiti Teknologi Malaysia	9
Lembaga Ilmu Pengetahuan Indonesia	8
Universitas Tadulako	8
Universitas Ahmad Dahlan	8
Gadjah Mada University	7
Bengkulu University	7
Telkom University	7
Universitas Negeri Surabaya	7
Universitas Islam Negeri Syarif Hidayatullah Jakarta	7
Universitas Swadaya Gunung Djati	6
Universiti Kebangsaan Malaysia	6
Utrecht University	6
Universitas Syiah Kuala	6
Federal Neuropsychiatric Hospital	5
Universitas Nusantara PGRI Kediri	5
Universitas Galuh	5
Universidade Federal do Rio de Janeiro	5
Kyung Hee University	5
University of Amsterdam	5
Universiti Utara Malaysia	5
Chiba University	5
Chinese University of Hong Kong	5
Wageningen University and Research Centre	5
Pecsi Tudomanyegyetem	5
Matej Bel University	5
Universidade do Estado do Rio de Janeiro	5
University of Zagreb	5
Baqiyatallah University of Medical Sciences	5
Akdeniz Universitesi	5
Indian Institute of Technology, Guwahati	5
Japan Advanced Institute of Science and Technology	5
Universidad Iberoamericana	5
Ankara Universitesi	5
Central University of Finance and Economics	5
King Saud University	5
Cumhuriyet Universitesi	5
Adekunle Ajasin University	5
University of Washington, Seattle	5
University of Wroclaw	5
Saint Mary׳s University	5
University of Ghana	5
Izmir Ekonomi Universitesi	5
Universidade de Coimbra	5
Russian Academy of Sciences	5
Razi University	5
Universidade Federal do Rio Grande do Norte	5
Universita Cattolica del Sacro Cuore	5
Universitatea Babes-Bolyai din Cluj-Napoca	5
Kanazawa University	5
University of Nigeria	5
Universidad de Granada	5
Curtin University	5
University of Nairobi	5
University of Karachi	5
University of Tartu	5
Universitas Indonesia	5
Institut Pertanian Bogor	5
Universitas Brawijaya	5
Universitas Sebelas Maret	5
South-West University Neofit Rilski	5
Indian Institute of Management Bangalore	5
Makerere University	5
University Malaysia Pahang	5
Universitas Kanjuruhan Malang	5
Universitas Muhammadiyah Sidoarjo	5
University of Science and Culture, Tehran	5
Pakuan University	5
Universitas Negeri Medan	5
Yayasan Salib Suci	4
Universitas Muhammadiyah Tangerang	4
Arizona State University	4
Tohoku University	4
Institute of Psychology of the Polish Academy of Sciences	4
Universitat Zurich	4
Uniwersytet Warszawski	4
Universidade Federal de Uberlandia	4
Monash University	4
Istanbul Universitesi	4
Pontificia Universidade Catolica do Rio de Janeiro	4
Universitas Diponegoro	4
Universitas Lampung	4
Universitas Pasundan	4
Universitas Terbuka	4
Instituto D׳Or de Pesquisa e Ensino	4
Universidade de Coimbra, Faculdade de Psicologia e de Ciencias da Educacao	4
Universitas PGRI Semarang	4
Universitas Serang Raya	3
PSTNT-BATAN	3
Universitas PGRI Palembang	3
Mõttemaru Oü	3
Nanyang Technological University	3
Kyoto University	3
Russian State University for the Humanities	3
Technische Universitat Dresden	3
Kumoh National Institute of Technology	3
Yildiz Technical University	3
The University of Warwick	3
University of Tokyo	3
SWPS Uniwersytet Humanistycznospołeczny	3
Constantine the Philosopher University	3
University of Mataram	3
Universitas Pattimura	3
Nisshin Seifun Group Inc	3
Universitas Negeri Jakarta	3
Universitas Negeri Padang	3
STKIP PGRI Sumbar	3
Politeknik Negeri Lhokseumawe	3
Universitas Islam Riau	3
Universitas Sarjanawiyata Tamansiswa	3
Universitas Muhammadiyah Purwokerto	3
International Development Center of	2
Politeknik Negeri Semarang	2
PTNBR-BATAN	2
Public Health Institute	2
Universitas Siliwangi	2
Arsari Group	2
MI Consulting Corporation	2
Institut Teknologi Sumatera	2
Universitas Borneo Tarakan	2
Guangdong Construction Polytechnic	2
Sekolah Tinggi Keguruan Dan Ilmu Pendidikan Pasundan	2
SMAN 2 Kota Sukabumi	2
Dinas Pendidikan	2
STKIP Bima	2
Universitas Singaperbangsa Karawang	2
Hanwha	2
Nagoya University	2
University of Illinois at Urbana-Champaign	2
Kangwon National University	2
Universiti Sains Malaysia	2
Universite Pierre et Marie Curie	2
Bar-Ilan University	2
Uniwersytet Opolski	2
Slovak Academy of Sciences	2
The University of British Columbia	2
University of Wollongong	2
Hokkaido University	2
International Islamic University Malaysia	2
Universidade da Madeira	2
Simon Fraser University	2
Institute for Materials Research, Tohoku University	2
National Science Museum, Tokyo	2
Baskent Universitesi	2
Universitas Udayana	2
Universitas Andalas	2
Satya Wacana Christian University	2
Institut Teknologi Sepuluh Nopember	2
University of the Philippines Los Banos	2
Japan International Cooperation Agency	2

Table 8 below describes twenty most-cited documents of Universitas Pendidikan Indonesia (UPI) in Scopus. The data will be useful resource for citation analysis on authors and its research pattern. Researchers can track journals or conferences with higher citations and plan it for future potential publication outlets. Citation to documents in Scopus, as previously described, has been integrated in ranking for both university and researcher. Thus, such data will be important for institutions to improve its competitiveness in research. University, for example, might use the data to inform authors on what subject to research or what journal to submit and soon. University can also encourage researchers to do more collaborative works, as those that have collaborators, especially foreign experts, tend to have better citations.

**Table 8:** Most-Cited Documents of Universitas Pendidikan Indonesia (UPI).

**Author(s)**	**Journal**	**Citation**
Vashaei et al. (2005)	Journal of Applied Physics	96
Cho et al. (2005)	Semiconductor Science and Technology	56
Topik, Yukawa, & Ito (2005)	Journal of Plant Research	46
Song et al. (2013)	Carbon	41
Ogi, Nandiyanto, & Okuyama (2014)	Advanced Powder Technology	37
Nandiyanto et al. (2013)	Chemical Engineering Science	31
Saito et al. (2006)	Journal of In-Service Education	29
Ray Hamidie et al. (2015)	Metabolism: Clinical and Experimental	26
Muraza et al. (2015)	Microporous and Mesoporous Materials	25
Arutanti et al. (2015)	ACS Applied Materials and Interfaces	25
Fatimah & Verhulst (2003)	Nonlinear Dynamics	25
Simonin et al. (2003)	Hydrometallurgy	23
Nandiyanto et al. (2013)	Langmuir	22
Aisyah et al. (2013)	Journal of Agricultural and Food Chemistry	21
Sundayana et al. (2017)	World Transactions on Engineering and Technology Education	20
Nandiyanto et al. (2014)	Chemical Engineering Journal	19
Arutanti et al. (2014)	AIChE Journal	19
Suhendi et al. (2013)	Langmuir	19
Radiman & Yuliani (2008)	Polymer International	19
Saito et al. (2007)	International Journal of Educational Development	19

## 2 Experimental design, materials, and methods

This data article collects the dataset from Scopus database. An institutional subscription is required to access the database. Once in Scopus database, institutional search was conducted using keyword “Indonesia”. From more than two hundred institutions, ten state educational universities were chosen. For each university, collection of data was conducted using search analysis tool. Documents per year, subject area of documents, source title of documents, documents type, country/territory, author name and number of documents, affiliation and most cited documents for each university were downloadable in .csv format. These data in .csv file were then collected in an .xlsx file for each university. Ten .xlsx files for ten Indonesian educational universities were then uploaded along with this data article.

Further usage of the data can be based on policy makers, researchers or stake holders’ point of view. For initial evaluation, a descriptive statistics using some tables and figures can be sufficient. However, a more detailed analysis will require further bibliometric analytic techniques and tools. Previous research can be guidance for researchers for a specific analysis on a university [4] or partial analysis of some universities as set of group [5] for Indonesian context.
